# Treatment and survival of non-metastatic rectal cancer in patients with inflammatory bowel disease: nationwide cohort study

**DOI:** 10.1093/bjsopen/zraf014

**Published:** 2025-03-25

**Authors:** Erik Lundqvist, Karin Westberg, Caroline E Dietrich, Åsa H Everhov, Pär Myrelid, Bengt Glimelius, Anna Martling, Caroline Nordenvall

**Affiliations:** Department of Surgery, Vrinnevi Hospital, Norrköping, Sweden; Department of Biomedical and Clinical Sciences, Faculty of Health, Linköping University, Linköping, Sweden; Division of Surgery, Danderyd Hospital, Stockholm, Sweden; Department of Molecular Medicine and Surgery, Karolinska Institutet, Stockholm, Sweden; Department of Medicine Solna, Clinical Epidemiology Division, Karolinska Institutet, Stockholm, Sweden; Department of Medicine Solna, Clinical Epidemiology Division, Karolinska Institutet, Stockholm, Sweden; Department of Clinical Science and Education, Södersjukhuset, Stockholm, Sweden; Department of Biomedical and Clinical Sciences, Faculty of Health, Linköping University, Linköping, Sweden; Department of Surgery, Linköping University Hospital, Linköping, Sweden; Department of Oncology and Pathology, Karolinska Institutet, Stockholm, Sweden; Department of Immunology, Genetics and Pathology, Uppsala University, Uppsala, Sweden; Department of Molecular Medicine and Surgery, Karolinska Institutet, Stockholm, Sweden; Department of Pelvic Cancer, Karolinska University Hospital, Stockholm, Sweden; Department of Molecular Medicine and Surgery, Karolinska Institutet, Stockholm, Sweden; Department of Pelvic Cancer, Karolinska University Hospital, Stockholm, Sweden

## Abstract

**Background:**

Patients with inflammatory bowel disease have an increased risk of colorectal cancer. There is a scarcity of large studies with a focus on rectal cancer in patients with inflammatory bowel disease. This study aimed to compare survival in resected patients with rectal cancer with and without inflammatory bowel disease.

**Methods:**

This national population-based study used the Colorectal Cancer Data Base. All Swedish patients ≥18 years of age with a diagnosis of stage I–III rectal cancer between 1997 and 2021, surgically treated with curative intent, were included and followed up until 2022. The outcome of interest was recurrence-free survival. Flexible parametric survival models adjusted for time since surgery, year of diagnosis, sex, age at diagnosis, and Charlson Co-morbidity Index were used to estimate proportional and time-dependent hazard ratios of recurrence-free survival with 95% confidence intervals.

**Results:**

Overall, 22 082 patients with rectal cancer were included, among whom 323 (1.5%) had inflammatory bowel disease. Neoadjuvant radiotherapy/chemoradiotherapy was given to 55% and 63% of patients with and without inflammatory bowel disease respectively. The median follow-up time was 5.2 years (interquartile range (i.q.r.) 2.3–10) in patients with inflammatory bowel disease and 5.9 years (i.q.r. 2.9–10) in patients without inflammatory bowel disease. Based on the adjusted proportional hazards model, no overall difference in recurrence-free survival was found (HR 1.05, 95% c.i. 0.87 to 1.26). In the time-dependent adjusted model, patients with rectal cancer with inflammatory bowel disease experienced a lower recurrence-free survival during the first year after surgery (1 year HR 1.36, 95% c.i. 1.06 to 1.73), after which there was no difference in comparison with patients without inflammatory bowel disease (5 years HR 0.77, 95% c.i. 0.56 to 1.06).

**Conclusion:**

Despite lower recurrence-free survival during the first year among those with inflammatory bowel disease, there were no long-term differences between patients with or without inflammatory bowel disease.

## Teaser

In this national cohort study of patients with rectal cancer and inflammatory bowel disease, the recurrence-free survival was time dependent. Despite lower recurrence-free survival during the first year among those with inflammatory bowel disease there were no long-term differences between patients with or without inflammatory bowel disease. Neoadjuvant radiotherapy/chemoradiotherapy was used to the same extent in both groups.

## Introduction

A diagnosis of inflammatory bowel disease (IBD) entails an increased risk of colorectal cancer, and the risk is directly related to age at IBD diagnosis and the duration of the disease^1–3^. Even if the risk patterns differ somewhat between Crohn’s disease and ulcerative colitis, patients with extensive proctocolitis, primary sclerosing cholangitis, male sex and a family history of colorectal cancer are especially predisposed to colorectal cancer development^4–6^. While studies have shown that the risk of colorectal cancer in patients with IBD has slowly decreased over time, there has been consistent evidence of worse survival in this group^7–9^.

Rectal cancer (RC) differs from colon cancer in terms of treatment principles. In RC, neoadjuvant treatment with radiotherapy alone or in combination with chemotherapy (CRT) is frequently used, whereas this is seldom provided in colon cancers. It has been reported that radiotherapy in RC patients with IBD and active inflammation should be used with caution due to a higher risk of toxicity^10^. A recent population-based study also reported that fewer patients with IBD with RC received radiotherapy^11^. The type of surgery chosen in RC patients with IBD is a question for debate, where international guidelines recommend proctocolectomy, with or without restoration with ileal pouch-anal anastomosis (IPAA), as the ‘standard’ procedure^12,13^.

Previous studies have explored the relationships in outcomes between IBD and colorectal cancer, but there are few studies with a focus on RC and with reliable stage and treatment information available^11,14^. Even though the treatment results in patients with RC have steadily improved^15^, it is still a disease with high morbidity rates and mortality rates.

The main aim of this study was to compare recurrence-free survival (RFS) in resected RC patients with and without IBD. Secondary aims were to describe the use of neoadjuvant and the type of surgical resection in patients with IBD, and the risk of metachronous colorectal cancer.

## Methods

This article was written in accordance with The Strengthening the Reporting of Observational Studies in Epidemiology (STROBE) checklist for the reporting of observational studies^16^. Ethical consent was obtained from the regional Ethics Committee in Stockholm (2014/71–31, 2018/328-32, 2021-00342 and 2023-03305-02).

### Data sources

This national population-based cohort study used data from the Colorectal Cancer Data Base (CRCBaSe)^17^. CRCBaSe is a multi-linked database between the Swedish Colorectal Cancer Register (SCRCR) and several national registers, including data from 109 000 index patients, recently updated to include patients up until 2021. The relevant registers for this study were the Swedish Cancer Register (SCR), the National Patient Register (NPR), the Total Population Register and the Cause of Death Register (CDR).

The SCRCR is a national quality-of-care register, with detailed clinical data on assessment, diagnosis, American Society of Anesthesiologists (ASA) classification, tumour level, neoadjuvant and adjuvant therapy, type of surgery and follow-up data (including recurrences) prospectively recorded on all patients with rectal adenocarcinoma in Sweden since 1995^18^. In its current form, it contains both clinical stage data according to the preoperative work-up and pathological stage data. The coverage is above 98% and the accuracy of reported data has been validated several times, proving good quality^19,20^.

The SCR was founded in 1958 and records 60 000 new malignant cases each year, including information about the histological type, localization and stage of the tumour. Reporting to the SCR is mandatory by law. For this study, the SCR was used to retrieve information about previous cancer diagnoses and to identify metachronous colorectal cancer or high-grade dysplasia (information available until 2021).

The NPR holds data on the date of admission, diagnosis and procedure codes from all inpatient-care episodes with national coverage since 1987, and all outpatient visits since 2001. The purpose of the register is to monitor the population’s health status over long intervals. Here, the NPR was used to identify patients with IBD according to the International Classification of Diseases (*Supplementary material*, *Table S1*), to receive information about co-morbidities as a means to calculate the Charlson Co-morbidity Index (CCI) and to verify previous surgical procedures using Nordic Medico-statistical Committee codes (*Supplementary material*, *Table S2*).

The CDR, founded in 1961, contains detailed information about the direct and indirect causes of death of all deceased Swedish citizens. The SCR, NPR and CDR registers are run by the National Board of Health and Welfare. The population register held by the Swedish Tax Agency forms the basis for the Total Population Register and contains demographic and socioeconomic data on all Swedish citizens.

### Study population

**Fig. 1:**
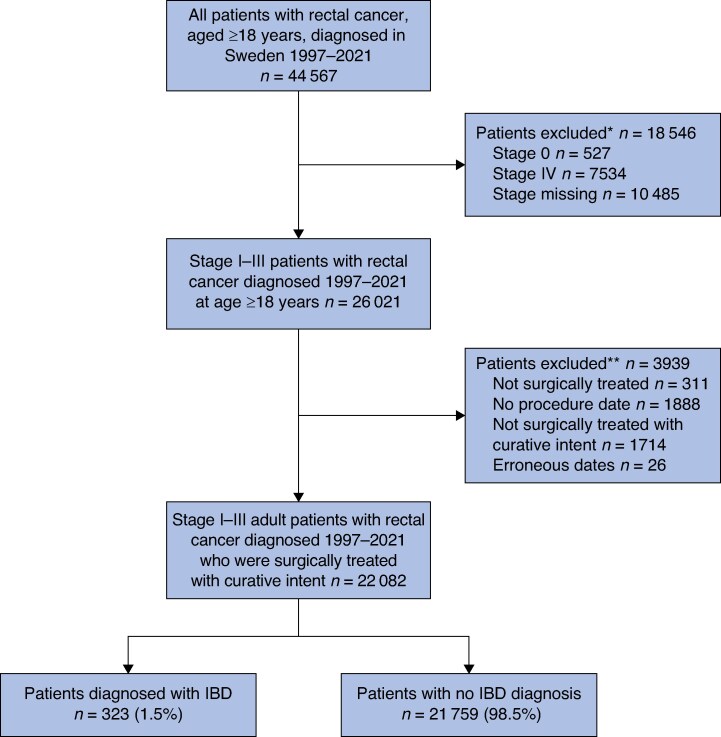
Flow chart showing the study cohort of Swedish stage I–III patients with rectal cancer diagnosed between 1997 and 2021 *Of these, 250 (1.4%) had IBD. **Of these, 105 (2.7%) had IBD. IBD, inflammatory bowel disease.

All patients in the CRCBaSe with a diagnosis of stage I–III RC between 1997 and 2021, aged ≥18 years and treated with abdominal surgery with curative intent (that is the surgeon estimated the surgery as curative), were included in this study. Patients who had distant metastases at the time of RC diagnosis and patients not treated with abdominal surgery with curative intent were excluded (*Fig. 1*). After exclusions, 22 082 patients remained and formed the study cohort for analyses.

### Exposure

Exposure of IBD was defined as having at least one diagnosis of Crohn’s disease, ulcerative colitis or IBD-unclassified (IBD-U) registered in the NPR before the RC diagnosis. If a patient had been diagnosed with both ulcerative colitis and Crohn’s disease, the most recent diagnosis before the RC diagnosis was used for classification of the subtype of IBD. All other patients were regarded as non-exposed to IBD.

### Outcomes of interest

The primary outcome of interest was RFS, defined as time to recurrence or death due to any cause, ignoring any second primary cancers. In addition, overall survival (OS), time to secondary dysplasia or metachronous tumour were investigated.

### Covariates

The selection of confounders was made *a priori* and included the following variables: age at RC diagnosis, year of RC diagnosis and co-morbidity level (CCI). CCI was calculated based on all diagnoses, excluding colorectal cancer and IBD, recorded within 5 (for the NPR) and 10 (for the SCR) years of the RC diagnosis, and divided into three categories: 0, 1 and ≥2^21^.

### Statistical analyses

Frequencies and percentages of patient and clinical characteristics were calculated. The proportions of categorical variables were compared using the Pearson chi-square test. For the main survival analysis, follow-up started on the date of RC surgery and ended on the date of recurrence, date of death, 10 years after surgery, date of migration or end of study interval (31 December 2022), whichever came first. In the analysis of OS, the date of recurrence was ignored. The underlying timescale was time since surgery throughout. The Kaplan–Meier method was used to calculate RFS and OS proportions in patients with and without IBD, compared using the log-rank test. Flexible parametric proportional hazards models (with 5 degrees of freedom for the baseline hazard rate) were used to estimate hazard ratios (HRs) with 95% confidence intervals (c.i.) comparing the composite outcome time to recurrence rate or death due to any cause (hereafter referred to as RFS^22^) between patients with or without a diagnosis of IBD. Both univariable and multivariable models were fitted. The multivariable model was adjusted for confounders (year of diagnosis, sex, age at diagnosis and CCI) and allowed for the effect of IBD to be time dependent (that is non-proportional hazards). Two degrees of freedom were used for the time-dependent effect. Year and age were modelled using restricted cubic splines (with 3 degrees of freedom) to avoid assuming a linear effect. In exploratory analyses, potential mediators were adjusted to assess how much of the effect could be explained by potential mediators (*Supplementary material, Tables S4* and *S5*). The data set was further stratified on the covariates stage and neoadjuvant treatment. A sensitivity analysis was also performed, where patients with at least two diagnoses of IBD before the RC diagnosis were considered exposed.

The incidence of metachronous colon cancer or dysplasia was further compared in patients with and without IBD. In this analysis, follow-up started at the time of surgery or 180 days after diagnosis of RC, whichever came last. Univariable and multivariable (adjusted as in the main analyses) proportional hazard models were fitted.

All statistical analyses were performed in Stata (Stata statistical software: release 18. Stata Corp LLC, College Station, TX, USA, 2019) using the merlin package^23^.

## Results

### Clinical characteristics

Overall, 323 (1.5%) of the 22 082 patients had IBD and among these, 83 (25.7%) had Crohn’s disease, 188 (58.2%) had ulcerative colitis and 52 (16.1%) had IBD-U. The proportion of patients with IBD in the study cohort was 1.1% before 2006 and 1.6% between 2006 and 2021 (*Table 1*). The proportion of female patients was 37.1% in patients with IBD and 40.1% in patients without IBD. The median age was 65 and 70 years respectively. A CCI of ≥1 was found in 26.0% of patients with IBD and in 20.0% of patients without IBD.

**Table 1:** Demographic and preoperative characteristics among stage I–III adult patients with rectal cancer diagnosed between 1997 and 2021, in patients with and without IBD

	IBD	No IBD	P*
Total	323 (1.5)	21 759 (98.5)	–
**Sex**			
Male	203 (62.9)	13 033 (59.9)	0.283
Female	120 (37.1)	8726 (40.1)	–
**Age (years) at diagnosis**			
18–49	54 (16.7)	1189 (5.5)	<0.001
50–59	61 (18.9)	2946 (13.5)	–
60–69	93 (28.8)	6425 (29.5)	–
70–79	70 (21.7)	7650 (35.2)	–
≥80	45 (13.9)	3549 (16.3)	–
Age (years), median (i.q.r.)	*65* (*53–74)*	*70* (*62–77)*	–
**Year of diagnosis**			
1997–2005	45 (1.1)	3996 (98.9)	0.123
2006–2013	134 (1.6)	8609 (98.4)	–
2014–2021	144 (1.6)	9154 (98.4)	–
**Charlson Co-morbidity Index**			
0	198 (61.3)	15 253 (70.1)	0.003
1	41 (12.7)	2146 (9.9)	–
2+	84 (26.0)	4360 (20.0)	–
**ASA classification**			
I–II	185 (57.3)	12 506 (57.5)	0.014
III–IV	79 (24.5)	4146 (19.0)	–
Missing	59 (18.3)	5107 (23.5)	–
**Previous colectomy**			
No	253 (78.3)	21 747 (99.9)	<0.001
Yes	70 (21.7)	12 (0.1)	–
*−* Ileorectal anastomosis	19 (5.9)	9 (0.0)	<0.001
**Tumour level**			
Low (0–5 cm)	120 (37.2)	6071 (27.9)	<0.001
Medium (6–10 cm)	117 (36.2)	8963 (41.2)	–
High (11–15 cm)	69 (21.4)	6413 (29.5)	–
Missing	17 (5.2)	312 (1.4)	–
**Clinical T stage**			
T1–T2	65 (20.1)	6569 (30.2)	0.002
T3	115 (35.6)	8522 (39.2)	–
T4	45 (13.9)	2520 (11.6)	–
TX/missing	98 (30.3)	5734 (26.4)	–
**Clinical N stage**			
N0	131 (40.6)	7786 (35.8)	0.055
N1–N2	99 (30.7)	8050 (37.0)	–
NX/missing	93 (28.8)	5923 (27.2)	–
**Incidental diagnosis of rectal cancer**			
Known RC diagnosis at surgery	296 (91.6)	21 139 (97.2)	<0.001
Unknown RC diagnosis at surgery	27 (8.4)	620 (2.8)	–

Values are *n* (%) unless otherwise indicated. Due to rounding, not all percentages sum up to 100%. *Calculated with Pearson chi-square test. IBD, inflammatory bowel disease; ASA, American Society of Anesthesiologists; i.q.r., interquartile range.

In 27 (8.4%) patients with IBD, the diagnosis date of RC was on or after the date of RC surgery. Among all patients with IBD, 70 (21.7%) had been operated on with colectomy before the diagnosis of RC, and of these, 19 (27.1%) had been reconstructed with ileorectal anastomosis (IRA). A tumour level below 6 cm from the anal verge was found in 120 (37.2%) patients with IBD and in 6071 (27.9%) patients without IBD.

### Stage and treatment of rectal cancer

Clinical pretreatment stage was registered in 230 (71.2%) patients with IBD and in 15 836 (72.8%) patients without IBD. Clinical T4-stage was found in 45 (19.6%) and 2520 (15.9%) patients with and without IBD (*Table 2*) and clinical N1–2-stage was found in 99 (43.0%) and 8050 (50.8%) respectively. A total of 179 (55.4%) patients with IBD and 13 660 (62.9%) patients without IBD, who later were resected with curative intention, received neoadjuvant treatment with either radiotherapy or chemoradiotherapy (*Table 2*). Clinical and pathological stages in patients with or without neoadjuvant RT are described in *Supplementary material*, *Table S3*. Among patients with neoadjuvant radiotherapy or chemoradiotherapy, clinical T4-stage was found in 37 (28.9%) patients with IBD and 2250 (22.2%) patients without IBD. After surgical resection, pathological T4-stage was found in 16 (12.5%) and 685 (6.8%) respectively. Clinical N1–2-stage was found in 76 (60.8%) and 6537 (66.4%) patients with and without IBD. Pathological N1–2-stage was found in 47 (37.6%) and 3927 (39.9%) patients with and without IBD. The overall proportion of pathological stage III was 38.7% in patients with IBD and 38.4% in patients without IBD.

**Table 2:** Clinical and treatment characteristics among stage I–III adult patients with rectal cancer diagnosed between 1997 and 2021, in patients with and without IBD

	IBD	No IBD	P*
	323 (1.5)	21 759 (98.5)	
**Neoadjuvant treatment**			
Radiotherapy only	132 (40.9)	10 435 (48.0)	0.031
Chemotherapy only	3 (0.9)	121 (0.6)	–
Chemoradiotherapy	44 (13.6)	3104 (14.3)	–
None	144 (44.6)	8099 (37.2)	–
**Type of surgery**			
Emergency	2 (0.6)	66 (0.3)	0.038
Elective	267 (82.7)	16 850 (77.4)	–
Missing	54 (16.7)	4843 (22.3)	–
**Type of procedure**			
Low anterior resection with/without deviating ileostomy	75 (23.2)	11 772 (54.1)	<0.001
Abdominoperineal excision with permanent colostomy	177 (54.8)	6975 (32.1)	–
Hartmann proctosigmoidectomy with colostomy	32 (9.9)	2522 (11.6)	–
Proctocolectomy with permanent ileostomy	26 (8.1)	76 (0.4)	–
Other	13 (4.0)	414 (1.9)	–
**Multiorgan resection**			
Yes	75 (23.2)	2765 (12.7)	<0.001
No	247 (76.5)	18 889 (86.8)	–
Missing	1 (0.3)	105 (0.5)	–
**Number of examined lymph nodes**			
< 12	74 (22.9)	5619 (25.8)	0.480
≥ 12	234 (72.5)	15 224 (70.0)	–
Missing	15 (4.6)	916 (4.2)	–
**Resection margins**			
R0	287 (88.9)	20 416 (93.8)	0.001
R1	25 (7.7)	853 (3.9)	–
R2	–	35 (0.0)	–
Missing	11 (3.4)	466 (2.1)	–
**pT stage**			
T0–T1	37 (11.5)	2126 (9.8)	0.003
T2	76 (23.5)	6491 (29.8)	–
T3	172 (53.3)	11 696 (53.75)	–
T4	32 (9.9)	1193 (5.5)	–
Missing	6 (1.9)	253 (1.2)	–
**pN stage**			
N0	197 (61.0)	13 398 (61.6)	0.961
N1–N2	119 (36.8)	7927 (36.4)	–
NX	7 (2.2)	434 (2.0)	–
**Pathological stage** ^†^			
I	100 (30.7)	6782 (31.2)	0.992
II	98 (30.3)	6632 (30.5)	–
III	125 (38.7)	8345 (38.4)	–
**Adjuvant chemotherapy**			
Yes	61 (18.9)	3259 (15.0)	0.051
No	262 (81.1)	18 500 (85.0)	–

Values are *n* (%) unless otherwise indicated. Due to rounding, not all percentages sum up to 100%. *Calculated with Pearson chi-square test. †Based on pathological TNM, but in a few missing cases (1–2%) based on clinical TNM. IBD, inflammatory bowel disease.

The proportion of patients with an emergency resection was low in both patients with (0.6%) and patients without IBD (0.3%) (*Table 2*). Of the 70 patients with IBD and previous subtotal colectomy, 60 (85.7%) underwent abdominoperineal excision (APE) and 10 had an anterior resection. In 253 patients with IBD without previous subtotal colectomy, 117 (46.2%) underwent APE, 70 (27.7%) low anterior resection, 31 (12.3%) Hartmann’s procedure and 26 (10.3%) proctocolectomy. Correspondingly, patients without IBD were surgically treated with low anterior resection in 54.1%, APE in 32.0%, Hartmann’s procedure in 11.6% and proctocolectomy in 0.3%. Microscopic non-radical resection margins (R1) were found in 7.7% of the patients with IBD and 3.9% of patients without IBD.

### Follow-up and survival

**Fig. 2:**
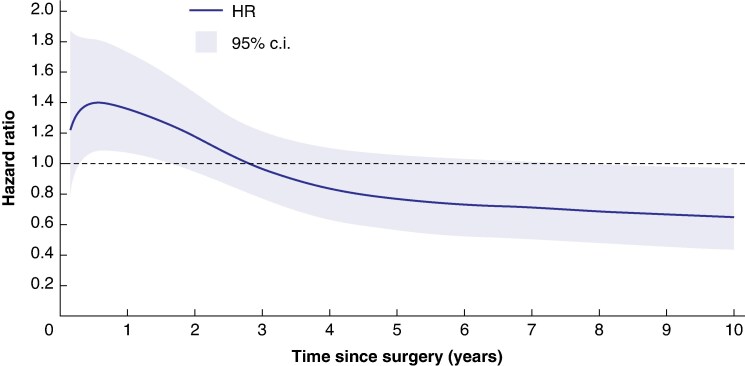
Time-varying hazard ratios (HRs) with 95% confidence intervals (c.i.) comparing recurrence-free survival* between patients with rectal cancer with IBD (blue line) and without IBD (dashed line) IBD was defined as having at least one diagnosis before rectal cancer diagnosis. Follow-up started at the date of surgery. Estimated from flexible parametric survival models adjusted for time since surgery, year of diagnosis, sex, age at diagnosis and CCI. *Time to the composite outcome of death due to any cause, or recurrence. IBD, inflammatory bowel disease; CCI, Charlson Co-morbidity Index; RFS, recurrence-free survival.

The median follow-up time was 5.2 years ( interquartile range (i.q.r.) 2.3–10) in patients with IBD and 5.9 years (i.q.r. 2.9–10) in patients without IBD. Among patients with IBD, 118 (36.4%) had a recurrence or died during follow-up, compared with 8710 (40.0%) in patients without IBD (*Table 3*). Kaplan–Meier estimates of RFS (*Supplementary material*, *Fig. S1*) showed no statistically significant difference (*P* = 0.552) between patients with and without IBD. Based on the adjusted proportional hazards model, no significant differences in RFS (HR 1.05, 95% c.i. 0.87 to 1.26) or OS were found (HR 1.10, 95% c.i. 0.91 to 1.33) (*Table 3*). However, when relaxing the proportional hazards assumption, the effect of IBD on RFS attenuated over time since surgery, with a 36% higher hazard rate at 1 year (HR_1 year_ 1.36, 95% c.i. 1.06 to 1.73), no difference at 5 years (HR_5years_ 0.77, 95% c.i. 0.56 to 1.06) and 35% lower at 10 years (HR_10years_ 0.65, 95% c.i. 0.43 to 0.97) (*Fig. 2*). Exploratory models, adjusting for neoadjuvant treatment and stage, resulted in similar estimates (*Supplementary material*, *Table S4*). In stratification on neoadjuvant treatment, the point estimate for RFS among those patients with IBD that did not receive any neoadjuvant treatment was 1.24 (95% c.i. 0.94 to 1.62), and 0.89 (95% c.i. 0.66 to 1.19) in patients that only received radiotherapy (*Supplementary material*, *Table S5*). A sensitivity analysis including only patients with IBD with at least two registered IBD diagnoses resulted in similar estimates. The cumulative local recurrence risk was 4.6% in patients with IBD and 3.6% among patients without IBD.

**Table 3:** Frequencies and hazard ratios (HRs) with 95% confidence intervals (c.i.) comparing recurrence or death during follow-up* (upper panel) and death during follow-up (lower panel) between patients with rectal cancer with and without IBD

IBD	Total	Recurrence or death during follow-up*	HR† (95% c.i.)	HR‡ (95% c.i.)
Yes	323 (100)	118 (36.5)	0.95 (0.79,1.14)	1.05 (0.87,1.26)
No	21 759 (100)	8710 (40.0)	1.00	1.00
		**Death during follow-up**	**HR† (95% c.i.)**	**HR‡ (95% c.i.)**
Yes	323 (100)	107 (33.1)	0.96 (0.79,1.16)	1.10 (0.91,1.33)
No	21 759 (100)	7903 (36.3)	1.00	1.00

Values are *n* (%) unless otherwise indicated. Follow-up started at the date of surgery. *Time to the composite outcome death due to any cause or recurrence. †Estimated from an unadjusted flexible parametric survival proportional hazards model. ‡Estimated from flexible parametric survival proportional hazards model adjusted for year of diagnosis, sex, diagnosis age and Charlson Co-morbidity Index. IBD, inflammatory bowel disease.

In the analyses of metachronous colorectal cancer or dysplasia, patients with a previous proctocolectomy (*n* = 166), who died (*n* = 470) or had a secondary colorectal cancer/dysplasia (*n* = 6) within 180 days after RC diagnosis or migrated (*n* = 553) before the start of follow-up were excluded, leaving a total of 20 887 patients. In patients with IBD, 2.0% (5 of 248) were diagnosed with a metachronous colorectal cancer or dysplasia 180 days or more after resection of the primary tumour (*Supplementary material*, *Table S6*). The corresponding percentage in patients without IBD was 2.0% (408 of 20 639) and after adjusting for confounders there was no statistically significant difference in incidence between the two groups (IBD *versus* non-IBD HR 1.22, 95% c.i. 0.50 to 2.95).

## Discussion

In this Swedish population-based study of more than 22 000 patients with non-metastasized RC, surgically resected with curative intent, there was overall no difference in prognosis between patients with and without IBD. Although HRs were similar over the whole follow-up time, patients with IBD had a worse outcome with a lower RFS at 1 year after RC surgery, and thereafter a similar RFS compared with patients without IBD.

Smaller case-control studies have reported similar survival between sporadic and IBD-associated RC^24,25^, indicating that advances in IBD treatment and surveillance protocols may positively impact the outcome of colorectal cancer. Though in line with a large British study, it is still concerning that the IBD-RC patients in the present study exhibited an increased risk of recurrence or death during the first year after diagnosis^11^. After the first year of follow-up, RFS returned to baseline in patients with IBD, and this improvement remained throughout the study interval. The reasons for this time-varying prognosis remain unknown, but it raises the suspicion of a different behaviour of IBD-associated colorectal cancer compared with sporadic colorectal cancer. The authors suggest future studies analyse the prognosis following diagnosis of colorectal cancer in patients with IBD with a time-varying approach.

There were signs of IBD-RC being different from sporadic RC. Previous studies have shown varying results regarding stage^7,11,14^, and in the present study, we found that close to 10% of patients with IBD had pT4 tumours, compared with 5.5% in patients without IBD. Biologically, the pathogenesis of IBD-associated colorectal cancer appears to be slightly different^26^, and there are several possible reasons for a more advanced pathological tumour stage in IBD: difficulties in a proper rectal examination due to stenosis or rigidity; areas of dysplasia may be multifocal and difficult to visually detect in a chronically inflamed rectum^27^; dysplasia may histologically be difficult to distinguish from inflammation^28^ and the sequence from dysplasia to manifest cancer may be shorter in IBD. It has also been proposed that it is more difficult to radiologically stage colorectal cancer in patients with IBD^29,30^. The authors found that in 8% of patients with IBD, RC diagnosis was not known before surgery, which may explain a larger proportion of non-radical resections.

Distal tumours were found in 37% of patients with IBD and 28% of patients without IBD. In ulcerative colitis, the reason for a distal tumour may be a more pronounced proctitis distally, causing an increased risk of dysplasia. This could also be true in patients with Crohn’s disease, in addition to perianal manifestations of the disease, sometimes impairing clinical evaluation through local inflammation and/or bowel stricture formation, potentially entailing an increased risk of malignancy^31^. A recent Swedish study found that in 24 patients with Crohn’s disease with postcolonoscopy colorectal cancer, 63% of the tumours were located in the most distal 5 cm of the rectum, suggesting the importance of digital rectal palpation and retroversion of the colonoscope in the rectum in patients with IBD^32^. It is known from previous RC studies that patients operated on for distal rectal tumours have a higher risk of local recurrence compared with those operated on for more proximal tumours^33–35^. Given the high proportion of distal tumours among patients with IBD, together with an almost 8% rate of non-radical resection margins, it would be reasonable to believe that the patients with IBD had a high risk of local tumour recurrence. Despite these risk factors for local recurrence in patients with IBD, there were no major differences in the actual numbers (4.6% and 3.6% in patients with IBD *versus* patients without IBD respectively).

More than half of the patients with IBD in our cohort received neoadjuvant treatment, which is substantially higher than in previous studies^11,36^. This result may be explained by the common use of short-course RT for intermediate-risk RC in Sweden, and that many Swedish patients have been involved in clinical RT studies^37^. Some previous studies have indicated an increased toxicity to chemoradiotherapy in patients with IBD, others have not^10,38^. With reservation that this study only included patients surgically resected with curative intent, neoadjuvant RT/CRT seemed to reduce the proportion of patients from clinical to pathological N1–N2 stage in 38% of patients with IBD and 40% of patients without IBD. However, clinical N-staging is difficult, with over-staging as the major problem^39^. Otherwise, the clinical stage together with the patient’s ability to tolerate a particular treatment decides the choice of treatment. During the explored interval, total neoadjuvant treatment was only provided to patients within trials and IBD was then an exclusion criterion^40^. Further studies emphasizing the treatment length, tolerance of and response to neoadjuvant treatment of RC in patients with IBD would be of value.

The optimal surgical strategy in patients with IBD with RC has been discussed^12,13^. The European Crohn’s and Colitis Organisation (ECCO) recommends proctocolectomy with or without IPAA in both patients with ulcerative colitis and Crohn’s disease in cases of colorectal cancer, while APE should be reserved for patients with very low RC. In the present study, only 10% (26 of 253) of patients with IBD without previous colorectal surgery were treated with proctocolectomy while 46% (117 of 253) underwent APE. Swedish IBD cancer surgery has not been centralized, and these patients are treated at centres doing RC surgery, but not always IBD surgery. The low number of proctocolectomies may be explained by treatment in centres with limited experience of IBD and cancer, and performing IPAAs. It underscores the importance of a multidisciplinary approach in patients with IBD with colorectal cancer, including gastroenterologists, clinical oncologists, radiologists, and colorectal surgeons with experience of IBD and RC^41^. Somewhat surprisingly, IBD was not associated with the risk of metachronous colorectal cancer. The option of segmental resection for colorectal cancer in patients with IBD highlights the importance of close surveillance with high-quality colonoscopy^32,42,43^.

The strengths of this study include its nationwide design and long study interval, allowing the inclusion of a large number of patients. In the SCRCR registry, data on recurrences have been documented to be accurate after 5 years of follow-up^44^, which most of the patients had. In the present study, there was no significant association between IBD and prognosis in the proportional hazards model. However, the time-dependent HRs revealed that the rate varied over time, which has not been shown in previous studies. Another strength of the SCRCR is the high-quality data of cTNM, pTNM and γpTNM, allowing for reliable analyses regarding tumour stage. The combination of the SCRCR with the NPR made it possible to adjust for co-morbidity, specify surgical procedures in detail and calculate the risk of metachronous colorectal cancers.

The study was limited by the number of patients with IBD. Despite this large national cohort including incident patients with RC spanning almost 25 years, the number of patients with IBD did not allow stratified analyses by type of IBD. Sensitivity analysis requiring two IBD diagnoses before RC surgery revealed similar results as the main analysis (data not shown). The proportion of patients with IBD was lower before 2006, which might reflect the absence of outpatient data before 2001. Another limitation was the exclusion of patients with missed procedure dates or not surgically treated with curative intent. Among these, we may have excluded patients with an initial curative intent who never proceeded to surgery. Lastly, the study lacked detailed data on neoadjuvant treatment (doses and length of treatment) and IBD characteristics such as the extent of inflammation and medical treatment. As in all observational studies, the risk of residual confounding should be considered when interpreting the results presented.

In this national study of patients surgically resected with curative intent for RC, the prognosis in patients with and without IBD was similar but time dependent. Patients with IBD experienced a worse prognosis initially, followed by a similar prognosis after the first year. More than half of the patients with and without IBD with RC received neoadjuvant RT. Despite the low use of proctocolectomies in patients with IBD with RC, there was no increased risk of metachronous high-grade dysplasia or colorectal cancer in these patients.

## Supplementary Material

zraf014_Supplementary_Data

## Data Availability

The data of CRCBaSe is stored securely at a guest server at the Karolinska Institutet, Stockholm, and can be demonstrated upon request to the corresponding author.
