# Supplementary material for: Treatment and survival of non-metastatic rectal cancer in patients with inflammatory bowel disease: nationwide cohort study
Source: BJS Open. 2025 Mar 25;9(2):zraf014. doi: 10.1093/bjsopen/zraf014 (PMC11934924; doi:10.1093/bjsopen/zraf014)
Supplement: zraf014_Supplementary_Data [file zraf014_supplementary_data.docx]

**Treatment and survival of non-metastatic rectal cancer in patients with inflammatory bowel disease – a nationwide cohort study**

Erik Lundqvist, M.D.^1 2^, Karin Westberg, M.D., Ph.D.^3 4^, Caroline E. Dietrich, Ph.D.^5^, Åsa H Everhov, M.D., Ph.D.^5 6^, Pär Myrelid, M.D., Ph.D.^2 7^, Bengt Glimelius, M.D., Ph.D.^8 9^, Anna Martling, M.D., Ph.D.^4 10^, Caroline Nordenvall, M.D., Ph.D.^4 10^

¹Department of Surgery, Vrinnevi Hospital, Norrköping, Sweden

²Department of Biomedical and Clinical Sciences, Faculty of Health, Linköping University, Linköping, Sweden

³Division of Surgery, Danderyd Hospital, Stockholm, Sweden

⁴Department of Molecular Medicine and Surgery, Karolinska Institutet, Stockholm, Sweden

⁵Department of Medicine Solna, Clinical Epidemiology Division, Karolinska Institutet, Stockholm, Sweden

⁶Department of Clinical Science and Education, Södersjukhuset, Stockholm, Sweden

⁷Department of Surgery, Linköping University Hospital, Linköping, Sweden

⁸Department of Oncology and Pathology, Karolinska Institutet, Stockholm, Sweden

⁹Department of Immunology, Genetics and Pathology, Uppsala University, Uppsala, Sweden

^10^Department of Pelvic Cancer, Karolinska University Hospital, Stockholm, Sweden

**Corresponding author.** Erik Lundqvist, Department of Biomedical and Clinical Sciences, Faculty of Health, Linköping University, Linköping, Sweden. phone: +46101042716, email-address: erik.lundqvist@liu.se **ORCID ID**; **0000-0001-5312-1023**

**Supplementary Materials - Index**

| **Supplementary Figures and Tables** |  | |
| --- | --- | --- |
| **SUPPLEMENTARY FIGURE 1** | ***pag. 3*** | |
| Recurrence-free survival proportions among stage I-III adult rectal cancer patients diagnosed 1997-2021, in patients with and without IBD. P-value from log rank test of difference in survival | | |
| **SUPPLEMENTARY TABLE 1** | ***pag. 4*** | |
| Diagnostic codes used to identify exposure of IBD, according to the international classification of diseases, version 7-10 | | |
| **SUPPLEMENTARY TABLE 2** | ***pag. 5*** | |
| All procedure codes for colectomy and reconstructive surgery | | |
| **SUPPLEMENTARY TABLE 3** | ***pag. 6*** | |
| Comparison of clinical and pathological T/N-stage among stage I-III adult patients with a RC diagnosis registered in the SCRCR 1997-2021, in patients with and without IBD and neoadjuvant (C)RT. | | |
| **SUPPLEMENTARY TABLE 4** | ***pag. 8*** | |
| Mediation by stage and neoadjuvant treatment , including stage and neoadjuvant treatment as mediators in model c, making the HR^c^s represent the direct effect of IBD not mediated through any of these. | | |
| **SUPPLEMENTARY TABLE 5** | ***pag. 9*** | |
| Adjusted hazard ratios (HRs) with 95% confidence intervals (CIs) comparing recurrence-free survival (RFS)* (upper panel) and overall survival (lower panel) between rectal cancer patients with and without IBD, by neoadjuvant treatment and stage. Follow-up started at date of surgery. | | |
| **SUPPLEMENTARY TABLE 6** | ***pag. 10*** | |
| Hazard ratios (HRs) with 95% confidence intervals (CIs) of secondary CRC and/or dysplasia in stage I-III adult rectal cancer patients with and without IBD. | | |
| **SUPPLEMENTARY TABLE 7** | | ***pag. 11*** |
| Proportions of patients with involved MRF on pre-operative imaging, whether exposed to IBD or not, diagnosed with rectal cancer 2006-2021. The variable was not available before 2006. | | |
| **SUPPLEMENTARY TABLE 8** | | ***pag. 12*** |
| Proportions of patients undergoing TME surgery for rectal cancer, whether exposed to IBD or not, diagnosed 2017-2021. | | |

**SUPPLEMENTARY FIGURE 1** Recurrence-free survival proportions among stage I-III adult rectal cancer patients diagnosed 1997-2021, in patients with and without IBD. P-value from log rank test of difference in survival

**
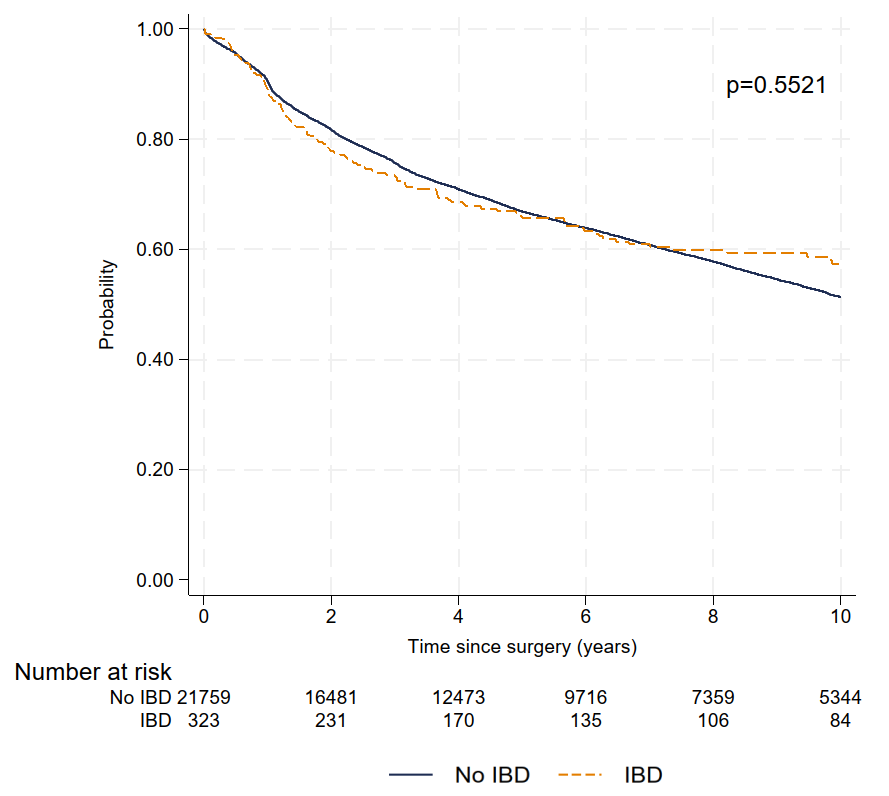
**

**SUPPLEMENTARY TABLE 1** Diagnosis codes used to identify exposure of IBD, according to the international classification of diseases, version 7-10

| **Diagnose** | **ICD codes** | | | |
| --- | --- | --- | --- | --- |
|  | **ICD7** | **ICD8** | **ICD9** | **ICD10** |
| CD | 572,00 ; 572,09 | 563,00 | 555 | K50 |
| UC | 572,20 ; 572,21 ; 578,03 ; | 563,10 ; 563,99 ; 569,02 | 556 | K51 |
| IBD-U |  |  |  | K52.3 |

*Abbreviations: ICD; International classification of diseases, CD; Crohn’s disease, UC; Ulcerative colitis, IBD-U; Inflammatory bowel disease – unclassified.*

**SUPPLEMENTARY TABLE 2** All procedure codes for colectomy and reconstructive surgery

| **Description** | **Nordic Medico-Statistical Committee (NOMESCO) Codes** | |
| --- | --- | --- |
|  | **<1997** | **≥1997** |
| Colectomy | 465 | JFH |
| Abdominoperineal excision |  | JGB3 |
| Low Anterior Resection |  | JGB0 |
| Hartman |  | JGB1 |
| Proctocolectomy |  | JFH20 or JFH+JGB50-61 |
| Reconstructive surgery   - Ileal-pouch anal anastomosis |  | JFH30, JFH31, JFH33, JGB50, JGB60, JGB61 |
| - Ileorectal anastomosis |  | JFH00, JFH01, JFC40, JFC41, JFG26, JFG29 |

**SUPPLEMENTARY TABLE 3** Comparison of clinical and pathological T/N-stage among stage I-III adult patients with a RC diagnosis registered in the SCRCR 1997-2021, in patients with and without IBD and neoadjuvant (C)RT (left panel: yes, right panel: no)

| **(a) Treated with neoadjuvant (C)RT** | | | | | **(b) Not treated with neoadjuvant (C)RT** | | | |  |
| --- | --- | --- | --- | --- | --- | --- | --- | --- | --- |
|  | ***T stage**** | **Clinical** | **Pathological** |  | | ***T stage**** | **Clinical** | **Pathological** | |
| **IBD** | *T1-T2* | 13 (10.2) | 34 (26.7) | **IBD** | | *T1-T2* | 52 (53.6) | 46 (47.4) | |
| (n=128) | *T3* | 78 (60.9) | 73 (57.0) | (n=97) | | *T3* | 37 (38.1) | 42 (43.3) | |
|  | *T4* | 37 (28.9) | 16 (12.5) |  | | *T4* | 8 (8.3) | 8 (8.2) | |
|  | *TX* | - | 5 (3.9) |  | | *TX* | - | <5 | |
|  |  |  |  |  | |  |  |  | |
| **No IBD** | *T1-T2* | 1 721 (17.0) | 3 476 (34.4) | **No IBD** | | *T1-T2* | 3 262 (55.2) | 2 900 (49.1) | |
| (n=10 115) | *T3* | 6 144 (60.7) | 5 765 (57.0) | (n=5 910) | | *T3* | 2 376 (40.2) | 2 656 (44.9) | |
|  | *T4* | 2 250 (22.2) | 685 (6.8) |  | | *T4* | 270 (4.6) | 290 (4.9) | |
|  | *TX* | - | 189 (1.9) |  | | *TX* | - | 64 (1.1) | |
| ***p-value*** |  | *0.049* | *0.011* |  | |  | *0.231* | *0.001* | |
|  |  |  |  |  | |  |  |  | |
|  | ***N stage***** |  |  |  | | ***N stage***** |  |  | |
| **IBD** | *N0* | 49 (39.2) | 74 (59.2) | **IBD** | | *N0* | 82 (78.1) | 70 (66.7) | |
| (n=125) | *N1-2* | 76 (60.8) | 47 (37.6) | (n=105) | | *N1-2* | 23 (21.9) | 32 (30.5) | |
|  | *NX* | - | <5 |  | | *NX* | - | <5 | |
|  |  |  |  |  | |  |  |  | |
| **No IBD** | *N0* | 3 307 (33.6) | 5 717 (58.1) | **No IBD** | | *N0* | 4 479 (74.6) | 3 931 (65.6) | |
| (n=9 844) | *N1-2* | 6 537 (66.4) | 3 927 (39.9) | (n=5 992) | | *N1-2* | 1 513 (25.3) | 1 827 (30.5) | |
|  | *NX* | - | 200 (2.0) |  | | *NX* | - | 234 (3.9) | |
| **p-value** |  | 0.188 | 0.288 |  | |  | 0.434 | 0.918 | |

** Among patients treated with neoadjuvant RT, 48 IBD patients and 3 424 non-IBD patients had cT stage TX/missing. Among patients not treated with RT, the corresponding numbers were 50 and 2 310.*

*** Among patients treated with RT, 51 IBD patients and 3 695 non-IBD patients had cN stage NX/missing. Among patients not treated with neoadjuvant RT, the corresponding numbers were 42 and 2 228.*

*Abbreviations:RC; rectal cancer, SCRCR; Swedish Colorectal Cancer Register, IBD; Inflammatory bowel disease, (C)RT; Radio- or Chemoradiotherapy.*

**SUPPLEMENTARY TABLE 4** Mediation by stage and neoadjuvant treatment, including stage and neoadjuvant treatment as mediators in model c, making the HR^c^s represent the direct effect of IBD not mediated through any of these.

| **Recurrence-free survival** | | | |
| --- | --- | --- | --- |
| **IBD** | **HR*^a^* (95% CI)** | **HR*^b^* (95% CI)** | **HR*^c^* (95% CI)** |
| Yes | 0.95 (0.79-1.14) | 1.05 (0.87-1.26) | 1.06 (0.89-1.26) |
| No | 1.00 | 1.00 | 1.00 |
| **All-cause mortality** | | | |
| **IBD** | **HR*^a^* (95% CI)** | **HR*^b^* (95% CI)** | **HR*^c^* (95% CI)** |
| Yes | 0.96 (0.79-1.16) | 1.10 (0.91-1.33) | 1.12 (0.93-1.36) |
| No | 1.00 | 1.00 | 1.00 |

***^a^*** *Estimated from an unadjusted flexible parametric survival proportional hazards model.*

***^b^*** *Estimated from flexible parametric survival proportional hazards model adjusted for year of diagnosis, sex, diagnosis age, and Charlson Comorbidity Index.*

***^c^*** *Estimated from flexible parametric survival proportional hazards model adjusted for year of diagnosis, sex, diagnosis age, Charlson Comorbidity Index, and mediating variables stage and neoadjuvant treatment.*

**SUPPLEMENTARY TABLE 5** Adjusted hazard ratios (HRs) with 95% confidence intervals (CIs) comparing recurrence-free survival (RFS)* (upper panel) and overall survival (lower panel) between rectal cancer patients with and without IBD, by neoadjuvant treatment and stage. Follow-up started at date of surgery.

| **Recurrence-free survival** | | | |
| --- | --- | --- | --- |
|  | *No neo* | *RT only* | *RT+CT or CT only* |
| **IBD** | **HR (95% CI)** | **HR (95% CI)** | **HR (95% CI)** |
| Yes | 1.24 (0.94-1.62) | 0.89 (0.66-1.19) | 1.30 (0.81-2.09) |
| No | 1.00 | 1.00 | 1.00 |
|  |  |  |  |
|  | *Stage I* | *Stage II* | *Stage III* |
| Yes | 0.90 (0.59-1.39) | 1.13 (0.83-1.55) | 1.07 (0.82-1.39) |
| No | 1.00 | 1.00 | 1.00 |
| **All-cause mortality** | | | |
|  | *No neo* | *RT only* | *RT+CT or CT only* |
| **IBD** | **HR (95% CI)** | **HR (95% CI)** | **HR (95% CI)** |
| Yes | 1.34 (1.01-1.77) | 0.87 (0.64-1.19) | 1.49 (0.88-2.49) |
| No | 1.00 | 1.00 | 1.00 |
|  |  |  |  |
|  | *Stage I* | *Stage II* | *Stage III* |
| Yes | 0.95 (0.61-1.47) | 1.05 (0.74-1.48) | 1.22 (0.93-1.59) |
| No | 1.00 | 1.00 | 1.00 |

*** Time to the composite outcome death due to any cause, or recurrence*.*

***^b^*** *Estimated from flexible parametric survival proportional hazards model adjusted for year of diagnosis, sex, diagnosis age, and Charlson Comorbidity Index, fully stratified by (1) neoadjuvant treatment, and (2) stage.*

**SUPPLEMENTARY TABLE 6** Hazard ratios (HRs) with 95% confidence intervals (CIs) of secondary CRC and/or dysplasia in stage I-III adult rectal cancer patients with and without IBD. Follow-up started at date of surgery or diagnosis +180 days, whichever came last. Patients with a previous proctocolectomy (n=166), who died (n=470), had a secondary CRC/dysplasia (n=6) within 180 days or migrated (n=553) were excluded

| **IBD** | **Total** | **Secondary CRC*** | **HR^a^ (95% CI)** | **HR^b^ (95% CI)** |
| --- | --- | --- | --- | --- |
| Yes | 248 (100) | 5 (2.0) | 1.09 (0.45-2.63) | 1.22 (0.50-2.95) |
| No | 20 639 (100) | 408 (2.0) | 1.00 | 1.00 |

** Including dysplasia.*

***^a^*** *Estimated from an unadjusted flexible parametric survival model.*

***^b^*** *Estimated from a flexible parametric survival model adjusted for time since surgery, year of diagnosis, sex, diagnosis age, and CCI.*

**SUPPLEMENTARY TABLE 7** Proportions of patients with involved MRF on pre-operative imaging, whether exposed to IBD or not, diagnosed with rectal cancer 2006-2021. The variable was not available before 2006.

| **MRF status** | **IBD**  **n=278** | **Non-IBD**  **n=17,763** | **p-value** |
| --- | --- | --- | --- |
| MRF negative, n (%) | 81 (29.1) | 5,971 (33.6) | 0.132 |
| MRF positive, n (%) | 17 (6.1) | 1,345 (7.6) |  |
| Missing, n (%) | 180 (64.7) | 10,447 (58.8) |  |

*Abbreviations: MRF; Mesorectal fascia, IBD; Inflammatory bowel disease*

**SUPPLEMENTARY TABLE 8** Proportions of patients undergoing TME surgery for rectal cancer, whether exposed to IBD or not, diagnosed 2017-2021. The variable was not available before 2017.

| **TME surgery** | **IBD**  **n=56** | **Non-IBD**  **n=5,365** | **p-value** |
| --- | --- | --- | --- |
| No TME, n (%) | 7 (12.5) | 1,027 (19.1) | 0.208 |
| TME, n (%) | 49 (87.5) | 4,338 (80.1) |  |
